# Pre-Exercise Hyperhydration-Induced Bodyweight Gain Does Not Alter Prolonged Treadmill Running Time-Trial Performance in Warm Ambient Conditions

**DOI:** 10.3390/nu4080949

**Published:** 2012-08-13

**Authors:** Pierre-Yves Gigou, Tommy Dion, Audrey Asselin, Felix Berrigan, Eric D. B. Goulet

**Affiliations:** 1 Research Centre on Aging, University of Sherbrooke, Sherbrooke, PQ J1H 4C4, Canada; Email: Pierre-Yves.Gigou@usherbrooke.ca (P.-Y.G.); Tommy.Dion@usherbrooke.ca (T.D.); Audrey.Asselin@usherbrooke.ca (A.A.); 2 Faculty of Physical Education and Sports, University of Sherbrooke, Sherbrooke, PQ J1K 2R1, Canada; Email: Felix.Berrigan@usherbrooke.ca

**Keywords:** hyperhydration, hydration, exercise, running, endurance performance, running economy

## Abstract

This study compared the effect of pre-exercise hyperhydration (PEH) and pre-exercise euhydration (PEE) upon treadmill running time-trial (TT) performance in the heat. Six highly trained runners or triathletes underwent two 18 km TT runs (~28 °C, 25%–30% RH) on a motorized treadmill, in a randomized, crossover fashion, while being euhydrated or after hyperhydration with 26 mL/kg bodyweight (BW) of a 130 mmol/L sodium solution. Subjects then ran four successive 4.5 km blocks alternating between 2.5 km at 1% and 2 km at 6% gradient, while drinking a total of 7 mL/kg BW of a 6% sports drink solution (Gatorade, USA). PEH increased BW by 1.00 ± 0.34 kg (*P* < 0.01) and, compared with PEE, reduced BW loss from 3.1% ± 0.3% (EUH) to 1.4% ± 0.4% (HYP) (*P* < 0.01) during exercise. Running TT time did not differ between groups (PEH: 85.6 ± 11.6 min; PEE: 85.3 ± 9.6 min, *P* = 0.82). Heart rate (5 ± 1 beats/min) and rectal (0.3 ± 0.1 °C) and body (0.2 ± 0.1 °C) temperatures of PEE were higher than those of PEH (*P* < 0.05). There was no significant difference in abdominal discomfort and perceived exertion or heat stress between groups. Our results suggest that pre-exercise sodium-induced hyperhydration of a magnitude of 1 L does not alter 80–90 min running TT performance under warm conditions in highly-trained runners drinking ~500 mL sports drink during exercise.

## 1. Introduction

It has been believed that exercise-induced bodyweight (BW) loss (EIBWL) of ≥2% impairs endurance performance (EP) during exercises conducted under temperate, warm and hot environmental temperatures [1,2]. However, under conditions of temperate and hot ambient temperatures Goulet [3] recently demonstrated through a meta-analysis that EIBWL ≤4% does not impair EP during laboratory-based cycling time-trials (TT) emulating real-world exercise conditions. Of particular importance is that none of the studies included in Goulet [3] showed a statistically significant impairment in cycling TT performance with EIBWL [4,5,6,7,8]. 

As surprising as it may be, only one laboratory-based study to date has looked at how EIBWL influences running performance. Fallowfield *et al.* [9] demonstrated that EIBWL of 2% decreased power output by 2.2% during a running test to exhaustion conducted at 70% maximal oxygen consumption (VO_2max_), 20 °C and 55% relative humidity (RH). This finding is hardly relevant for competitive runners whose racing goal is to run a fixed distance as fast as possible. Moreover, studies have shown that exercise intensity during racing conditions never remains constant but rather constantly varies throughout either on a macro- or micro-scale [10,11]. Finding an answer to how EIBWL impacts running TT performance is imperative in order to widen the evidence base and improve fluid intake guidelines. 

Several recent field studies have observed a significant relationship between EIBWL and running EP, with the fastest athletes showing the greatest loss in BW [12,13,14]. Such findings are not easy to explain, but a suggested possibility is that EIBWL could improve running economy, such that athletes who lose the greatest BW are those that best optimize their running speed and, hence, EP [15]. Such assertion makes sense given that there is a strong association between running economy and distance running performance and that this variable is a better predictor of EP than VO_2max_ in elite runners with similar VO_2max_ [16]. 

Despite the proposed benefits of maintaining EIBWL loss <2% during exercise, real-life competitions are associated with high relative and absolute speeds preventing competitive distance runners from drinking a sufficient volume of fluid to maintain adequate hydration [17]. For example, highly-trained runners have been demonstrated to consume ~150–300 mL/h of fluid and lose more than 2% BW during 15 to 21 km competitive runs [18,19]. Instead of attempting to increase fluid intake during exercise, it could be wiser for runners to hyperhydrate before exercise. In fact, in addition to potentially delaying or preventing EIBWL ≥2%, this technique has been demonstrated to improve cardiovascular and thermoregulatory functions [20] and increase cycling endurance capacity [21], compared with starting an exercise euhydrated. One potential drawback of PEH, however, is that the extra fluid load to be carried could impair running economy and hinder EP, although Beis *et al.* [22] recently showed that PEH does not alter running economy during a 30 min run conducted at a low intensity.

This study compared the effect of PEH and PEE in highly-trained runners during an 18 km running TT performed in warm temperature and comprising 8 km at 6% gradient (480 m of vertical climbing). If indeed PEH-associated gain in BW reduces running economy and consequently EP, it is believed that such a protocol would capture it, at least during the inclined parts of the run. We hypothesized that in highly-trained runners the hyperhydration-associated gain in BW would not be sufficient to significantly impact running speed, would improve cardiovascular and thermoregulatory functions and, under an exercise situation where athletes can adjust their speed according to body cues and knowledge of the distance completed, would not provide a hydration-related performance advantage, compared with PEE.

## 2. Methods

### 2.1. Subjects

Six non-heat-acclimatized, highly-trained competitive male athletes agreed to participate in this study. Among them, three were marathon runners, two were long-distance triathletes (Half-Ironman™ and Ironman™ distance) and one was a short-distance triathlete (Olympic distance). Their mean (±SD) age, height, BW, % fat mass (FM), % fat-free mass (FFM), maximal heart rate and peak oxygen consumption (VO_2peak_) were 31 ± 7 years, 179 ± 7 cm, 78 ± 10 kg, 11% ± 4%, 89% ± 4%, 192 ± 9 beats/min and 69 ± 3 mL/kg/min, respectively. Subjects were tested over the winter and early spring months of 2011 and were in the preparation phase of their training. The procedures and risks of the study were explained to the six volunteers and informed written consent was obtained. Since the subjects could not be blinded to the treatments they received, neither the specific goals of the study nor the hypotheses tested were explained. All procedures were approved by the University of Sherbrooke Institutional Review Board.

### 2.2. Overview of the Study

**Figure 1:**
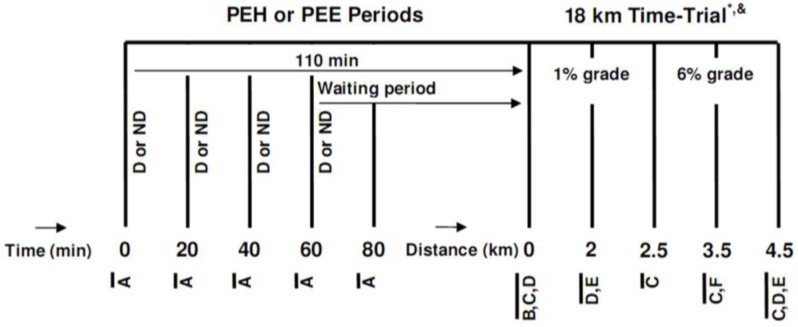
Schematic representation of the research protocol. (D or ND) Drink or no drink; (**A**) Measurement of urine volume, urine specific gravity, bodyweight, heart rate, perceived thirst, abdominal bloating and pain, nausea and dizziness; (**B**) Measurement of urine volume, urine specific gravity and bodyweight; (**C**) Measurement of rectal temperature, skin temperature and heart rate; (**D**) Measurement of perceived exertion, perceived thirst, perceived heat stress and abdominal discomfort; (**E**) Consumption of 1 mL/kg bodyweight of sports drink; (**F**) Measurement of perceived exertion and perceived thirst; PEH: Pre-exercise hyperhydration; PEE: Pre-exercise euhydration; * this 4.5 km block was repeated four times; ^&^ after running 9 km, subjects stopped for measurement of urine volume, urine specific gravity and bodyweight and to consume one sports gel.

After a preliminary visit and a familiarization phase, subjects underwent two experimental trials, started in either a hyperhydrated or euhydrated state, which were conducted in a randomized, crossover fashion, 7–10 days apart, at the same time of the day. After their arrival at the laboratory, participants either passively waited (PEE) or hyperhydrated (PEH) during a 110 min period, after which they underwent an 18 km running TT (comprising a total of 480 m of vertical climbing) on a motorized treadmill at an ambient temperature of ~28 °C and 25%–30% RH. A distance of 18 km was chosen since it was estimated that the course would be completed in ~80 to 90 min, which is the typical time well-trained runners require to complete a half-marathon. The TT was performed under an ambient temperature of 28 °C since no study had yet evaluated the effect of EIBWL upon EP at this temperature. A schematic description of the research protocol is presented in Figure 1.

### 2.3. Preliminary Testing

Four to seven days before the familiarization trial, subjects underwent a measurement of height, BW, body composition, VO_2peak_ and maximal heart rate. Height was determined to the nearest 0.5 cm with a wall stadiometer and with subjects wearing only socks. Bodyweight was measured in the nude and post-void to the nearest 100 g with a digital scale (Seca 707, Seca, Germany). Fat mass and FFM were measured using dual-energy X-ray absorptiometry technology (Lunar Prodigy, GE Healthcare, USA). Peak VO_2_ was measured on a motorized treadmill using an Oxycon Pro (Jaeger, Germany) expired gas analysis system that had been automatically calibrated with gases of known concentration. After subjects had warmed-up for 10–15 min at a self-selected pace and 1% gradient, treadmill speed was adjusted to 10 km/h with a speed increment of 1 km/h/min until 15 km/h, followed by a 2% gradient increment/min until volitional exhaustion of subjects.

### 2.4. Pre-Experimental Protocol

Over the study period (21–27 days), subjects were allowed to continue their training routine but refrained from any physical activity and diuretic substances such as alcohol and caffeine 24 h prior to the three running trials (familiarization run and two experimental runs). Lower leg strength training and dietary supplement intake were forbidden for 48 h prior to the trials. For the last 24 h prior to the familiarization trial, subjects kept and filled a fluid and diet log, which were replicated over the last 24 h prior to the experimental runs. Subjects went to sleep at the same time of the night prior to the running trials. Prior to bedtime and 90 min before their arrival at the laboratory before each trial, subjects consumed 500 mL water. In order to ensure a similar nutritional and hormonal state prior to the trials, subject drank a 240 kcal, 237 mL nutritional drink (Boost^®^, Nestlé, Switzerland) 120 min before reporting to the laboratory. After the drink had been consumed, subjects remained fasted (except for water intake) until the start of the running trials. 

### 2.5. Familiarization Trial

Seven to ten days prior to the first experiment a familiarization trial was conducted to minimize any learning effect, familiarize subjects with the measurement techniques and optimize subjects’ pacing strategy for the upcoming two experimental trials. Subjects were required to run as fast as possible during an 18 km TT conducted under the same ambient temperature, RH and wind speed, while wearing the same experimental equipments, clothes and running shoes, drinking the same sports drink, eating the same energy gel, running the same course, listening to the same music and following the same experimental procedures as during the two forthcoming experimental runs. 

### 2.6. Pre-Exercise Hyperhydration and Euhydration Periods

Upon arrival at the laboratory, subjects provided a midstream urine sample for urine specific gravity assessment (PAL-10S, Atago, USA), voided their bladder completely (graduated urinal), were weighed in the nude with a high precision scale (Bx-300+, Atron Systems, USA) and instrumented with a T-31 Polar electrode (Polar USA, USA). Following the measurement of heart rate after a 2–3 min seated rest period, subjects rated on a scale of 1 (none) to 5 (extreme) different subjective parameters (perceived thirst, abdominal bloating and pain, nausea and dizziness). Then the 110 min long PEE or PEH period began. No fluid was given to subjects during PEE. During PEH, subjects drank a total of 26 mL fluid/kg BW of a 130 mmol/L (7.5 g NaCl), 4 °C aspartame-flavored (5 g/L) (Crystal Light, Kraftfoods, USA) sodium solution, provided at a rate of 6.5 mL/kg BW every 20 min for the first 60 min. The design of the PEH protocol (total fluid volume, length, rate of ingestion, fluid temperature) was inspired by that previously used by Goulet *et al.* [21], which was associated with no untoward side-effects. Subjects were required to drink each volume of fluid within 5 min to standardize the time between each urine collection and weighting period. Heart rate, subjective perceptions, urine volume (graduated urinal), urine specific gravity and equipment-corrected BW were sequentially measured in the 18th, 38th, 58th, 78th and 108th min. Subjects were instrumented with the skin probes between the 40th and 80th min, whereas the rectal probe was installed in the 80th min. The changes in BW from before to after the PEE and PEH periods were taken as a reflection of the changes in body water status. Insensible water loss was not measured and was assumed to be similar between trials. A sodium solution was used to induce hyperhydration since the use of glycerol had been banned by the WADA in January 2010 [23]. Results of a pilot study conducted in our laboratory in two highly-trained subjects showed that a 130 mmol/L sodium solution was well-tolerated and produced levels of hyperhydration equivalent to those of glycerol-induced hyperhydration [24].

### 2.7. Eighteen Kilometer Time-Trial

The 18 km TT run, starting from the 110th minute, consisted of four successive, 4.5 km blocks alternating between 2.5 km at 1% [25] and 2 km at 6% gradient performed on a calibrated motorized treadmill (TMX 22, Trackmaster, USA) at ~28 °C (DVTH, Supco, USA) and 25%–30% RH (psychometric chart). To simulate radiant heat stress, two, 500-watts halogen lights (Workshop, Globe electric Company, QC, Canada) were placed ~50 cm above and ~20 cm behind the subject’s head. Before the start of exercise and at the end of each running block, measures of rectal temperature, skin temperature, heart rate, perceived exertion (Borg scale, 20-point scale, 6: very, very light; 20: very, very hard), perceived thirst (11-point scale, 1: none; 11: extreme), perceived heat stress (7-point scale, 1: none; 7: extreme) and abdominal discomfort (5-point scale, 1: none; 5: extreme) were taken. Moreover, for each block, measures of rectal and skin temperatures and heart rate were taken at 2.5 km and 3.5 km, perceived exertion and thirst at 2 km and 3.5 km and abdominal discomfort and perceived heat stress at 2 km. During the runs, subjects received continuous fan-cooling (wind speed of 200 m/min), consumed 1 mL/kg BW of a 6% sports drinks solution (Gatorade, Pepsico, USA) at 2 km and 4.5 km of each block, for a total of 7 mL/kg BW, were encouraged throughout, and were made aware of the distance completed but not of their running speed. To help understand the relationship between BW loss and running speed, subjects were required to stop running for 8 min at 9 km, during which time they were removed from the treadmill, voided their bladder, toweled dry, ate one 110 (27 g carbohydrates (CHO)) kcal energy gel (CarbBoom, Carb Boom Sports Nutrition, Canada) and were weighed while holding the disconnected cables tight to their chest. In addition to these procedures, the 8 min long resting period was necessary for the subjects to reach a respiratory rate that allows a valid measurement of BW and to disconnect and reconnect the skin probes from and to the switch box. At the end of the runs, subjects quickly stepped off the treadmill, voided their bladders, toweled dry and their BW was again measured. Subjects then rapidly removed the rectal and skin probes, running shoes, clothes and Polar electrode worn during the runs and a final nude BW was taken. Finally, a measurement of room temperature and RH was taken.

### 2.8. Bodyweight

Upon arrival at the laboratory and at the end of the running TTs, the running shoes, clothes and equipment worn by subjects during the PEE and PEH periods as well as during the 18 km TT were weighed using a digital compact scale (Symmetry, Cole Parmer, USA). The weight of the tape used to hold the probe cables and that of the energy gel were also measured. Hence, when necessary, measurement of BW was carefully corrected to take into account any excess weight. At 9 km, BW was corrected for half the sweat trapped in the subjects’ clothes, running shoes and tape measured at the end of the running TTs. 

### 2.9. Heart Rate and Rectal, Skin and Body Temperatures

Heart rate was measured continuously using a Vantage NV Polar heart rate monitor (Polar USA, USA). Rectal temperature was measured with a YSI 401 rectal probe (Yellow Springs Instrument, USA) inserted 10-cm beyond the anal sphincter and securely held in place with the aid of a lightweight harness developed in our laboratory. Skin temperature was measured with YSI 409 B probes (Yellow Springs Instrument, USA) placed on the left side of the body at the leg, chest and arm level and held in place with Transpore tape (3M, USA). Mean skin and body temperatures were determined as suggested by Grucza *et al.* [26]. The rectal and skin probes were connected to a switch box linked to a high precision digital thermometer (Traceable 4005, Control Company, USA).

### 2.10. Sweat Loss

Sweat loss was computed using the change in BW from the post PEE or PEH period to the post TT period and was corrected for the weight of the energy gel, fluid intake and urine loss during exercise. No correction was made for insensible water loss and the loss of mass associated with the respiratory exchange of O_2_ and CO_2_, and all were assumed to be similar between TTs.

### 2.11. Percent Bodyweight Loss

Percent BW loss was computed using the following formula: 


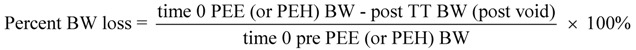


### 2.12. Statistical Analysis

The key outcome variable in this study was the difference in TT performance between interventions. On the basis of an estimated CV of 1.5% for the 18 km TT [27,28], a power analysis (α = 0.05, β = 0.2) revealed that six subjects would be sufficient to detect a 2.5% change in TT performance. Data were tested for normality of distribution using the Shapiro-Wilk test and analyzed using either paired sample *t*-tests and one- or two-way (treatment × time) repeated measures analyses of variance (ANOVA). Sphericity was verified and, if violated, a Greenhouse-Geisser correction was applied. Significance was defined as *P* < 0.05. Data reported in the text are expressed as means ± SD, and for sake of clarity, those in figures as means ± SEM. Analyses were performed with Microsoft Office Excel 2003 (version 11.8341.8341) and SPSS (version 12.0.0) softwares.

## 3. Results

### 3.1. Laboratory Temperature and Relative Humidity

For both ambient temperature and RH, a time effect was observed between groups over the course of the study period. Specifically, ambient temperature increased from 27.6 ± 0.1 °C at the onset of the PEE and PEH periods to 27.7 ± 0.2 °C (*P* = 0.02) at the end of the TTs, whereas the RH changed from 25% ± 2% to 30% ± 2% (*P* < 0.01) over this time period. No trial or interaction effect was observed.

### 3.2. Pre-Exercise Hyperhydration and Euhydration Periods

#### 3.2.1. State of Hydration of Subjects at the Arrival at the Laboratory

Subjects were adequately and similarly hydrated when they arrived at the laboratory for both trials. This is supported by non-significant differences observed between PEE and PEH with respect to urine specific gravity (1.014 ± 0.010 *vs.* 1.014 ± 0.004 g/mL, *P* = 0.97), BW (78.4 ± 9.3 *vs.* 78.3 ± 9.4 kg, *P* = 0.49), urine production (189 ± 60 *vs.* 130 ± 98 mL, *P* = 0.53) and heart rate (60 ± 11 *vs.* 56 ± 10 beats/min, *P* = 0.47). 

#### 3.2.2. Fluid Balance

**Figure 2:**
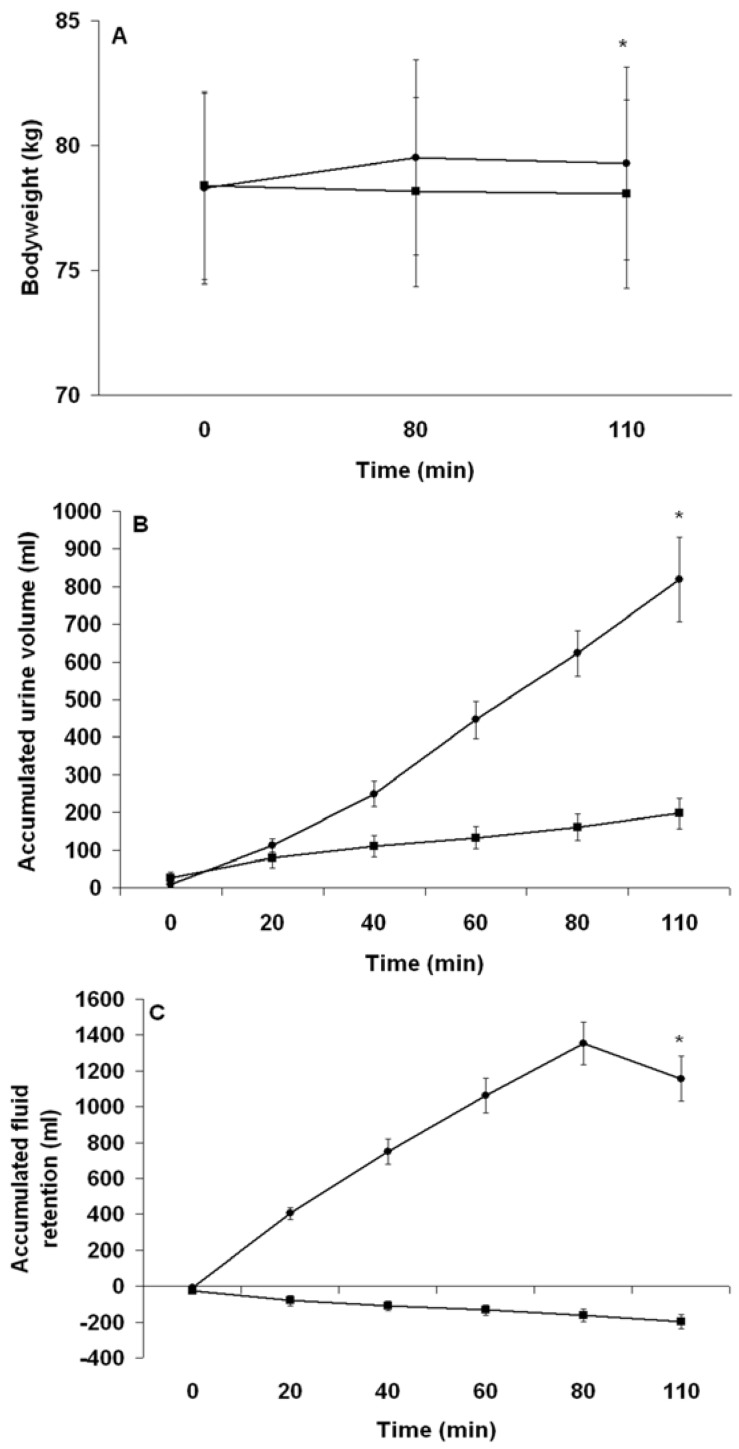
Change in bodyweight (**A**) and accumulated urine production (**B**) and fluid retention (**C**) during the pre-exercise euhydration (■) and hyperhydration (●) periods. * Significant trial effect. Results are mean ± SEM.

During PEH, subjects ingested 1976 ± 294 mL of aspartame-flavored fluid, together with 5.9 ± 0.9 g of sodium. Due to the time constraints imposed at each drinking point, one subject could not ingest more than 23 mL/kg BW of the sodium-containing fluid, and another subject could not ingest more than 24 mL/kg BW of the sodium-containing fluid. Figure 2A demonstrates the changes in BW from before to after the PEE and PEH periods, whereas Figure 2B,C shows the accumulated urine volume and fluid retention throughout the PEE and PEH periods, respectively. There was a significant interaction effect in the BW changes (−0.34 ± 0.12 *vs.* 1.00 ± 0.34 kg, *P* < 0.01), accumulated urine volume (−198 ± 101 *vs.* 819 ± 273 mL, *P* < 0.01), and fluid retention (−198 ± 101 mL, −2.52 ± 1.26 mL/kg BW *vs.* 1156 ± 309 mL, 14.81 ± 3.32 mL/kg BW, *P* < 0.01) between PEE and PEH. Urine specific gravity was significantly lower during PEH (1.005 ± 0.003 g/mL, indicative of well-hydrated state) than PEE (1.020 ± 0.006 g/mL, indicative of euhydrated state) (*P* < 0.05) immediately before starting the TTs.

#### 3.2.3. Heart Rate and Perceptual Responses

Heart rate decreased over time (*P* = 0.04), but no trial or interaction (*P* = 0.75) effect between groups was observed. An interaction (*P* < 0.01) effect was observed for the change in perceived thirst between trials, with thirst increasing over time with PEE but decreasing with PEH. Subjective feelings of dizziness, nausea and abdominal bloating and pain were not significantly different between groups (*P* > 0.05).

### 3.3. Time-Trial Periods

#### 3.3.1. Performance

**Figure 3:**
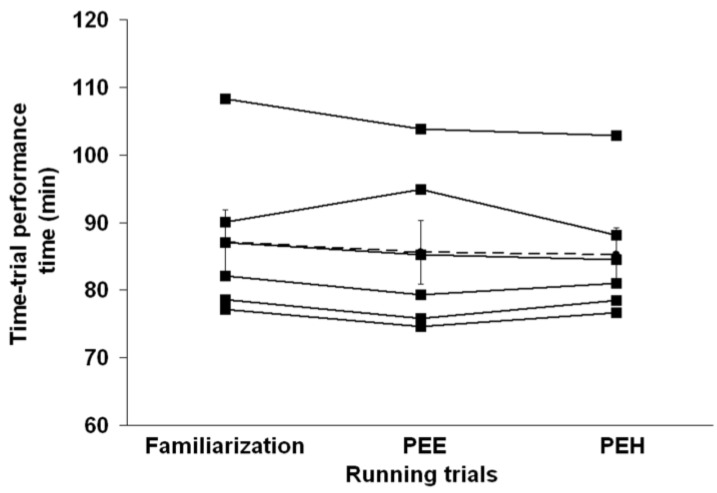
Individual time-trial performance time during the familiarization period and with pre-exercise euhydration (PEE) and hyperhydration (PEH). The dashed line represents the mean (±SEM) time-trial performance time observed in each of the three running trials.

**Figure 4:**
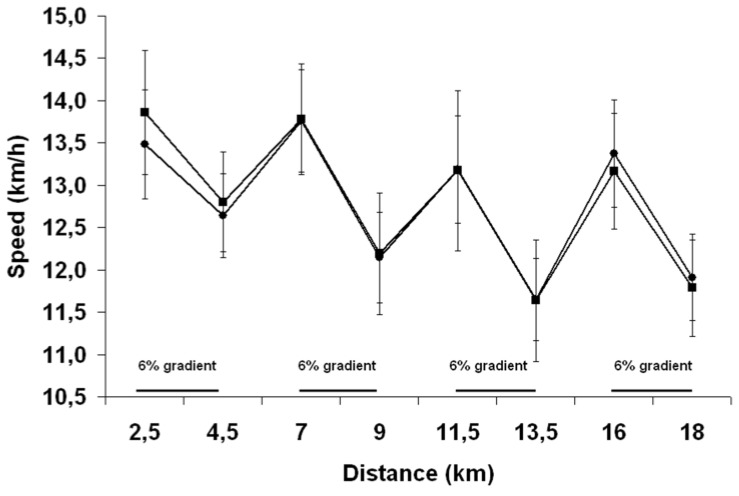
Change in running speed throughout the run with pre-exercise euhydration (■) and hyperhydration (●). Results are mean ± SEM.

There was no trial order effect (*P* = 0.82), indicating that there was no learning effect from Trial 1 to Trial 2. Moreover, no order effect was observed when the familiarization trial was taken into account (*P* = 0.28). The TT performance time CV from the familiarization phase to Trial 1 and from Trial 2 to Trial 3 was 2.0% and 2.7%, respectively. There was no difference in the time that the subjects took to complete the 18 km TT (PEE: 85.6 ± 11.6 min; PEH: 85.3 ± 9.6 min, *P* = 0.82). The first 9 km were completed in 41.5 ± 5.1 min for PEE and 41.8 ± 4.4 min for PEH, respectively (*P* = 0.64), compared with 44.1 ± 6.8 min for PEE and 43.5 ± 5.3 min for PEH (*P* = 0.49) for the last 9 km. Figure 3 reports the individual TT performance times observed during the familiarization period and with PEE and PEH. No difference in performance was observed between the familiarization trial (87.20 ± 11.48 min) and the PEE and PEH trials (*P* = 0.18). As depicted in Figure 4, there was a time effect (*P* < 0.01) but no trial effect (*P* = 0.91) or interaction effect (*P* = 0.45) between groups in running speed throughout the TT. No trial, time or interaction effects between groups were detected regarding the running speed maintained by subjects during the flat portion of the run. However, during the inclined portion of the run, a time effect (*P* = 0.02) was observed between groups but no trial effect or interaction effect was observed.

#### 3.3.2. Fluid Balance

Subjects consumed a total volume of 547 ± 69 mL of sports drink for an hourly drinking rate of 403 ± 70 *vs.* 401 ± 61 mL/h with PEE and PEH, respectively. Total CHO consumption was 60 ± 4 g, with a total of 44 ± 6 and 44 ± 5 g/h for PEE and PEH, respectively. Total sweat loss and hourly sweat rate (PEE: 2642 ± 426 mL, 1878 ± 372 mL/h; PEH: 2652 ± 433 mL, 1876 ± 318 mL/h) did not differ significantly between trials. No time effect, trial effect (*P* = 0.11) or interaction effect between groups was observed in the change in urine production during exercise (PEE: 30 ± 12 mL; PEH: 73 ± 51 mL). 

**Figure 5:**
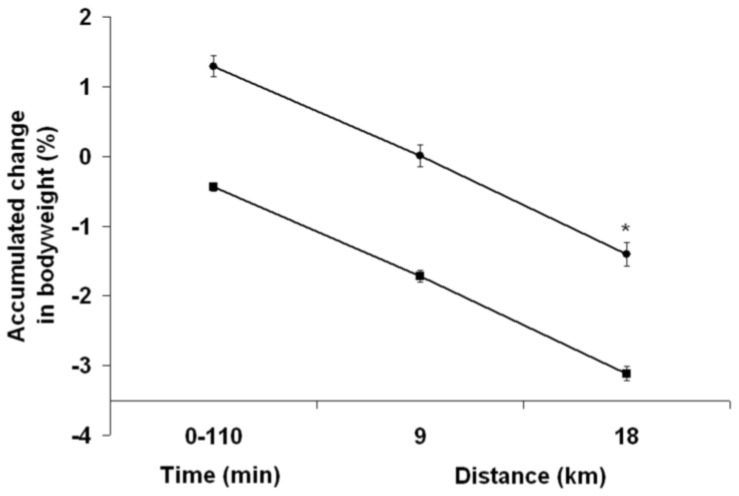
Accumulated change in bodyweight from the start to the end of the time-trial with pre-exercise euhydration (■) and hyperhydration (●). * Significant trial effect. Results are mean ± SEM.

As shown in Figure 5, there were a time effect (*P* < 0.01) and trial effect (*P* < 0.01) but no interaction effect between groups regarding the cumulated change in BW from the start of the PEE and PEH periods to the end of the TT. With PEH, a relative loss of BW at the end of the TT amounted to 1.4% ± 0.4%, whereas with PEE this loss reached 3.1% ± 0.3% (*P* < 0.01). After the first 9 km, subjects had lost 1.7% ± 0.2% of their BW with PEE, compared to 0.00% ± 0.4% with PEH (*P* < 0.01). At the 9 km mark, the difference in BW between groups was 1.22 ± 0.35 kg (*P* < 0.01), whereas at the end of the TT the difference was 1.19 ± 0.31 kg (*P* < 0.01). When the loss of BW associated with the waiting period before exercise start was discarded, TT-induced BW loss reached 2.6% ± 0.2% with PEE, which is still significantly more important than PEH (*P* < 0.01). During exercise, urine specific gravity increased more with PEE than with PEH (*P* = 0.02), with final values of 1.025 ± 0.003 *vs.* 1.020 ± 0.005 g/mL, respectively.

#### 3.3.3. Heart Rate

**Figure 6:**
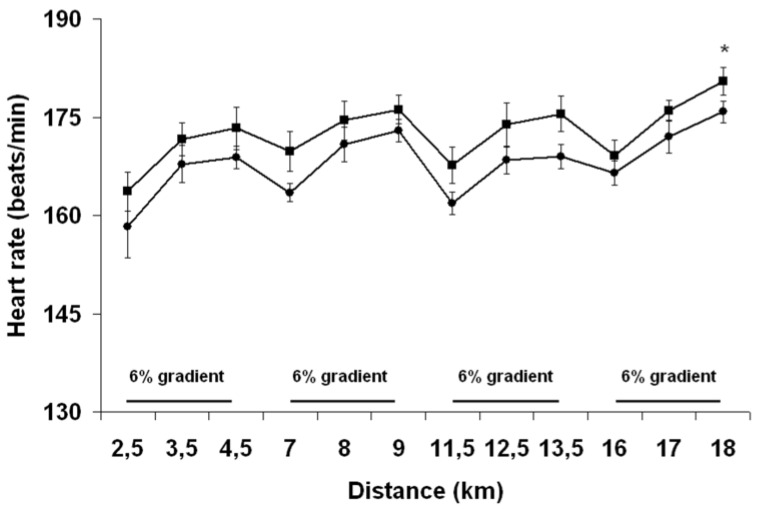
Change in heart rate throughout the run with pre-exercise euhydration (■) and hyperhydration (●). * Significant trial effect. Results are mean ± SEM.

As depicted in Figure 6, heart rate was significantly lower (−5 ± 1 beats/min) with PEH than PEE throughout the TT, with a time effect (*P* < 0.01) and group effect (*P* = 0.03) observed during both the flat and inclined portions of the run. Subjects exercised at a lower percent of maximal heart rate during PEH than PEE (88% ± 5% *vs.* 90% ± 6%, *P* = 0.01).

#### 3.3.4. Rectal and Mean Body and Skin Temperature

**Figure 7:**
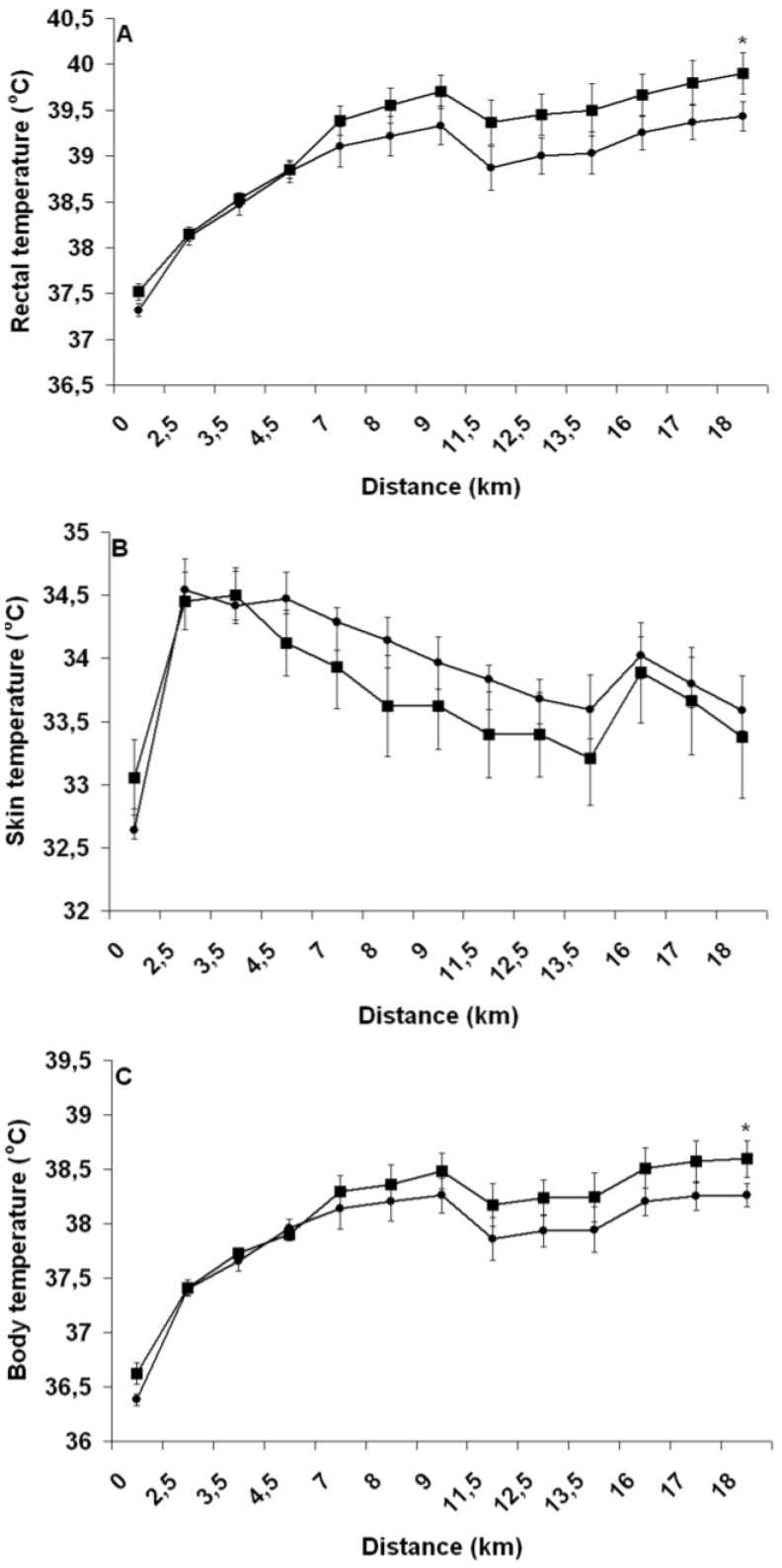
Change in rectal (**A**) and mean skin (**B**) and body (**C**) temperature throughout the run with pre-exercise euhydration (■) and hyperhydration (●). * Significant trial effect. Results are mean ± SEM.

As shown in Figure 7, there was a significant time effect (all *P* < 0.01) in the change in rectal (A) and mean skin (B) and body (C) temperatures during the TT. A significant trial effect was observed for the change in rectal (*P* < 0.01) and mean body (*P* = 0.04) temperature, with PEH reducing thermal stress more than PEE. On average, PEH decreased rectal and mean body temperature during the TT by 0.3 ± 0.1 and 0.2 ± 0.1 °C, respectively. No trial effect (*P* = 0.39) was observed for the change in mean skin temperature. A significant interaction effect was observed in the change in rectal temperature (*P* < 0.01) across time between trials, but no such effects were observed with regard to mean skin (*P* = 0.42) and body temperature (*P* = 0.06).

#### 3.3.5. Perceptual Responses

**Figure 8:**
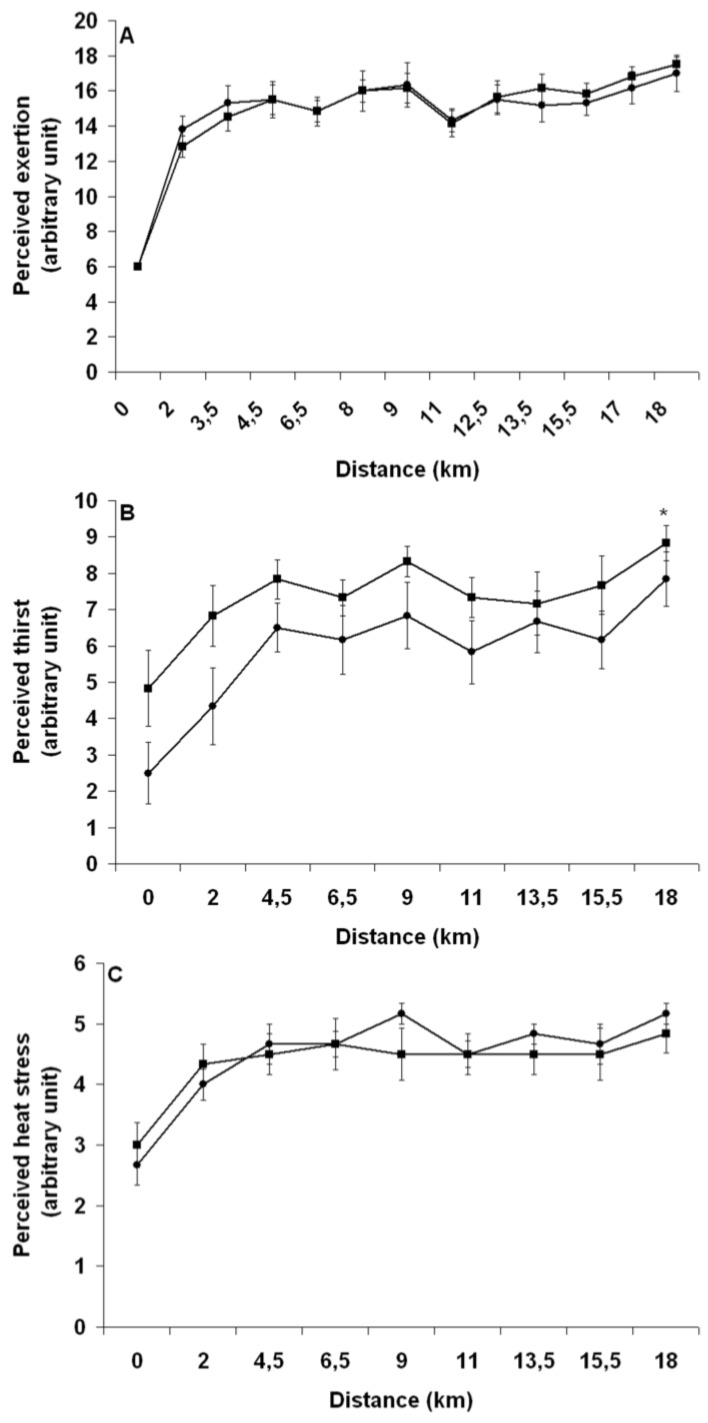
Change in perceived exertion (**A**), thirst (**B**) and heat stress (**C**) throughout the run with pre-exercise euhydration (■) and hyperhydration (●). * Significant trial effect. Results are mean ± SEM.

Figure 8 depicts the changes in perceived exertion (A), thirst (B) and heat stress (C) over the course of the TT with PEE and PEH. In all cases, a time effect (*P* < 0.01) was observed. Perceived thirst was significantly lower with PEH than with PEE (*P* < 0.01). There was no difference in perceived exertion (*P* = 0.89) and heat stress (*P* = 0.57) between groups. For both PEE and PEH, feeling of abdominal discomfort increased significantly over time with a mean starting and final value of 1.3 ± 0.2 and 2.2 ± 0.3 units (*P* = 0.01), but no trial (*P* = 0.17) or interaction effect between groups was observed.

## 4. Discussion

To the best of our knowledge, this is the first study to examine the impact of PEH upon running TT performance. Perhaps even more importantly, this is the first time that a study examines how EIBWL *per se* influences prolonged running TT performance. The goal of PEH is to maximize EP by preventing a ≥2% EIBWL. Since running economy is closely associated with running EP but PEH can potentially impair this variable, the findings of this study are therefore relevant for runners. Several novel and valuable findings were obtained in the current study. For highly-trained runners who drink ~500 mL of fluid during an 80–90 min intense run, the results suggest that (1) PEH does not contribute to improved running EP although it prevents >2% EIBWL; and (2) a PEH-induced BW gain of 1 kg does not impact running speed. Finally, our results suggest that a 130 mmol/L sodium solution could prove as efficient as glycerol in preventing diuresis-induced fluid loss.

It has generally been accepted that ≥2% EIBWL systematically hinders EP regardless of whether the exercise is conducted under temperate, warm or hot temperatures [1]. In the only study conducted using running as a means to test the impact of EIBWL on performance, Fallowfield *et al.* [9] demonstrated that subjects with a <2% EIBWL took longer to reach exhaustion than those with 2% EIBWL. Goulet *et al.* [21] demonstrated that, compared with PEE, glycerol-induced hyperhydration that was sufficient to prevent >2% EIBWL significantly increased endurance capacity during an ~12 min long incremental cycling test to exhaustion following 2 h of steady-state cycling.

Ebert *et al.* [29] showed that, despite reducing the metabolic cost of exercise, EIBWL of ~2 kg decreased hill-climb time-to-exhaustion following 2 h of fixed-intensity cycling exercise, compared with a well-hydrated state. Results of the current study clearly contradict those of the aforementioned studies. These discrepant findings between studies can be reconciled on the basis that the three studies showing a positive effect of hydration used fixed-exercise intensity to exhaustion exercise protocols, whereas the current study used a TT performance protocol. In fact, from a statistical point of view, no study to this day has been able to demonstrate that an EIBWL of ≤4% BW impairs EP, compared with a well-hydrated state [4,5,6,7,8]. In a recent meta-analysis, Goulet [3] even demonstrated that EIBWL improves (+0.06%), albeit not significantly, rather than decreases cycling EP. The present study adds a new finding to the literature in showing that attempting to prevent ≥2% EIBWL through the use of PEH is unlikely to confer an increase in running TT performance.

Some runners are reluctant to use PEH as they worry about losing speed due to the extra amount of BW needing to be carried. One way through which PEH could potentially reduce running speed is by increasing the O_2_ cost of running for a given speed. In fact, the relationship between running economy and performance has been well documented, with many studies demonstrating a strong relationship between running economy and distance running performance [30,31]. However, Armstrong *et al*. [32] demonstrated that the maintenance of euhydration did not increase O_2_ cost during 10 min of running at 70% and 85% VO_2max_ in highly trained runners, compared with a 5.5% BW loss (2.6 kg) induced by water deprivation. Recently, and of more relevance to the current study, Beis *et al.* [22] showed that a 0.9 kg hyperhydration BW gain induced by creatine and glycerol did not alter running economy in trained runners completing a 30 min run at 60% VO_2max_ under ambient temperatures of 10 °C and 35 °C. An important limitation of those studies is that no measure of EP was taken. Our findings therefore extend those of the actual literature and suggest that carrying an additional water load of 1 kg is unlikely to interfere with running speed and presumably not decrease running economy in trained athletes, both under flat and hilly conditions.

Up until 2010, the year when WADA banned glycerol due to its possible masking effect [23], glycerol-induced hyperhydration was the technique of choice used by those athletes wanting to start exercise with an extra fluid reservoir. In fact, in comparison with water-induced hyperhydration, the addition of glycerol (1–1.2 g/kg BW) to a large fluid load (20–26 mL/kg BW) had been demonstrated to increase fluid retention by ~1000 mL, or 13 mL/kg BW, during ~135 min long hyperhydration protocols [24]. Results of the present study suggest that a 130 mmol/L sodium solution ingested at a volume of 26 mL/kg BW provides a fluid retention capability that compares favorably well with the now banned glycerol-induced hyperhydration technique. This effect was likely due to the hypertonic nature of the hyperhydration solution (~330 mOsmol/kg H_2_O) which, in the face of an accrued body water, likely allowed a slight increase in or at least prevented the excessive decrease of the production of ADH. Hence this enabled a significant reduction of the subsequent rate of water excretion at the kidney level, compared to a situation where only tap water had been ingested. How long could the present PEH protocol sustain a marked increase in body water at rest is not clear. Interestingly, Sharon *et al*. [33] have demonstrated that the ingestion of 26 mL/kg BW of a 80 mEq/L NaCl solution over 2 h maintains hyperhydration for 4 h, compared with water- and glycerol-induced hyperhydration which sustains hyperhydration for 3 h and 5 h, respectively. Despite these promising findings, further studies directly comparing glycerol- and sodium-induced hyperhydration are needed before any recommendations can confidently be made to athletes. 

Pre-exercise hyperhydration reduced both heart rate and rectal temperature during exercise, which is in agreement with results of most studies that have compared the effect of PEE and PEH upon physiological functions during exercises ≥45 min [20]. Increased cardiovascular and thermoregulatory strain has been believed to be key causal mechanisms explaining the EIBWL-associated reduction in EP [1]. Clearly, the greater cardiovascular and thermoregulatory challenges encountered by athletes during PEE did not perturb their running ability. Moreover, perceived exertion was not more elevated with PEE, suggesting that these physiological perturbations were not sensed by the brain as adding significant strain to an already severely stressed body. Since no study has demonstrated a deleterious effect of EIBWL upon EP, it must be that the aforementioned relationship between thermoregulatory and cardiovascular strain and EP was established from results of studies that used fixed-intensity exercise protocols. To this effect, Atkinson *et al.* [34] have argued that when exercise work rate is self-selected, there is evidence to suggest that the pacing strategy, or the “selection” of work rate by athletes, is regulated specifically to ensure that factors that are classically implicated as causing fatigue are instead regulated so that they do not adversely affect physiological variables before the known endpoint of exercise is reached. During fixed-intensity testing protocols where the end-point of exercise is unknown, thereby depriving the brain of a key anchor point, and where the body cannot appropriately deal with physiological insults, commands from the brain could be sent to working muscles to prematurely stop exercising in order to prevent a catastrophic failure from happening [35]. Our findings suggest that despite EIBWL alters the internal milieu in an “unfavorable” manner, the implication and physiological relevance of such changes during running TT performance are likely insignificant, at least in our studied population and within ≤3% EIBWL. 

A possible limitation of the present study relates to its small sample size. However, based on a predicted TT CV of 1%, 1.5% and 2%, a conventional power analysis reveals that 67, 147 and 259 participants, respectively, would have been needed to detect the difference in EP time (0.35%) observed between trials in the current study. Obviously, it would have been impossible to recruit that many highly-trained runners to participate in the study and, from a financial point of view, to test that number of subjects. In order to precisely track changes in BW during the TT, subjects were required to stop running at 9 km. We do not believe that this confounded our findings since running speed remained the same between groups from 7 km to 13.5 km and the pattern of changes in heart rate and rectal temperature between groups was also not altered after 9 km. 

## 5. Conclusions

In conclusion, our findings suggest that, although pre-exercise sodium-induced hyperhydration of a magnitude of 1 L and sufficient to prevent >2% EIBWL decreases cardiovascular and thermoregulatory stress, it does not alter 80–90 min running TT performance in highly-trained runners. Further studies using a larger sample size are needed to confirm the present findings.
